# Rituximab and obinutuzumab differentially hijack the B cell receptor and NOTCH1 signaling pathways

**DOI:** 10.1016/j.isci.2021.102089

**Published:** 2021-01-22

**Authors:** Jennifer Edelmann, Arran D. Dokal, Emma Vilventhraraja, Karlheinz Holzmann, David Britton, Tetyana Klymenko, Hartmut Döhner, Mark Cragg, Andrejs Braun, Pedro Cutillas, John G. Gribben

**Affiliations:** 1 Centre for Haemato-Oncology, Barts Cancer Institute, Queen Mary University of London, Charterhouse Square, London, EC1M 6BQ, UK; 2 Department of Internal Medicine III, Ulm University, Albert-Einstein-Allee 23, 89081 Ulm, Germany; 3 Kinomica Limited, Biohub Alderley Park, Alderley Edge, Macclesfield, Cheshire, SK10 4TG, UK; 4 Center for Clinical Research, Genomics Core Facility, Ulm University, Helmholtzstr. 8/1, 89081 Ulm, Germany; 5 Sheffield Hallam University, City Campus, Howard Street, Sheffield, S1 1WB, UK; 6 Antibody and Vaccine Group, Centre for Cancer Immunology, University of Southampton Faculty of Medicine, Tremona Road, Southampton, SO16 6YD, UK

**Keywords:** immunology, systems biology: proteomics, cancer

## Abstract

The anti-CD20 monoclonal antibodies rituximab and obinutuzumab differ in their mechanisms of action, with obinutuzumab evoking greater direct B cell death. To characterize the signaling processes responsible for improved B cell killing by obinutuzumab, we undertook a phosphoproteomics approach and demonstrate that rituximab and obinutuzumab differentially activate pathways downstream of the B cell receptor. Although both antibodies induce strong ERK and MYC activation sufficient to promote cell-cycle arrest and B cell death, obinutuzumab exceeds rituximab in supporting apoptosis induction by means of aberrant SYK phosphorylation. In contrast, rituximab elicits stronger anti-apoptotic signals by activating AKT, by impairing pro-apoptotic BAD, and by releasing membrane-bound NOTCH1 to up-regulate pro-survival target genes. As a consequence, rituximab appears to reinforce BCL2-mediated apoptosis resistance. The unexpected complexity and differences by which rituximab and obinutuzumab interfere with signaling pathways essential for lymphoma pathogenesis and treatment provide important impetus to optimize and personalize the application of different anti-CD20 treatments.

## Highlights

• Rituximab and obinutuzumab induce strong ERK and MYC activation
• Obinutuzumab supports apoptosis induction by aberrant SYK phosphorylation
• Rituximab elicits stronger anti-apoptotic signals by activating AKT
• Rituximab elicits stronger pro-survival signals by enhancing NOTCH1 signaling

## Graphical Abstract

**Figure:**
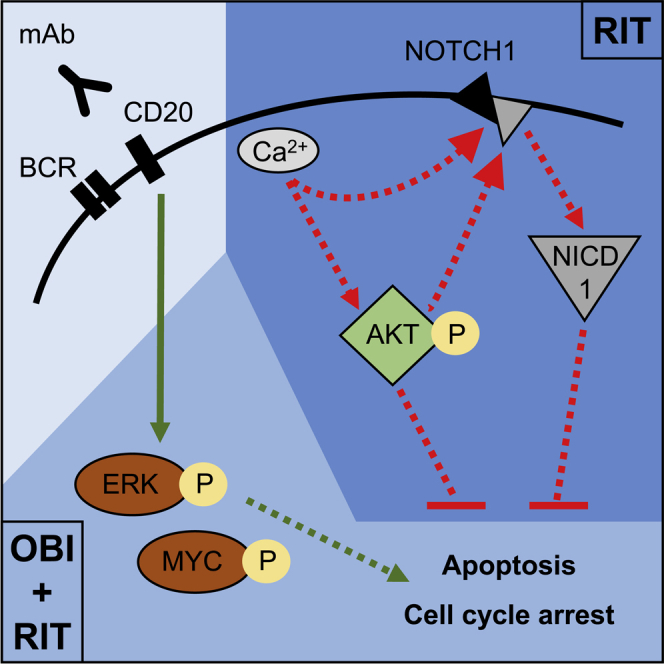

## Introduction

Combination of the anti-CD20 monoclonal antibody rituximab with chemotherapy has significantly improved outcomes for patients with CD20^+^ B cell lymphoma (Marshall et al., 2017). Despite this success, the mechanisms of action of rituximab remain incompletely understood, largely because they are manifold and encompass low levels of direct B cell killing next to immune-mediated effects (Marshall et al., 2017). The latter are mainly mediated by complement recruitment, but they also comprise antibody-dependent cellular cytotoxicity and phagocytosis (Marshall et al., 2017; Rouge et al., 2020). The clinical success of rituximab has fostered the development of novel anti-CD20 antibodies such as obinutuzumab with stronger capacity for direct B cell killing and a glycoengineered Fc-fragment for improved effector cell recruitment. Higher efficiency in the induction of direct B cell death was achieved by introducing a sequence alteration into the elbow-hinge region of the monoclonal antibody, rendering it more Type II, and so less able to cluster CD20 in the membrane, reducing complement-dependent cytotoxicity when compared with rituximab and other Type I monoclonal antibodies (Mossner et al., 2010). The molecular effects that this alteration has on B cell signaling are relatively undocumented. A non-apoptotic lysosomal form of cell death has been shown for obinutuzumab (Alduaij et al., 2011), whereas the limited degree of rituximab-induced cell death has been associated with apoptosis following increased B cell receptor (BCR) signaling (Walshe et al., 2008; Franke et al., 2011; Pavlasova et al., 2018).

Phase III clinical trials comparing rituximab and obinutuzumab head-to-head demonstrated superiority of obinutuzumab over rituximab in the treatment of chronic lymphocytic leukemia (CLL; CLL11 trial, ClinicalTrials.gov ID: NCT01010061) and follicular lymphoma (GALLIUM trial, ClinicalTrials.gov ID: NCT01332968) with regard to minimal residual disease negativity, progression-free survival (PFS), and in the case of CLL, overall survival (Goede et al., 2014, 2015; Marcus et al., 2017). However, in first-line treatment of diffuse large B cell lymphoma (DLBCL; GOYA trial; ClinicalTrials.gov ID: NCT01287741), obinutuzumab failed to show benefit over rituximab (Vitolo et al., 2017). The reasons for non-superiority of obinutuzumab in DLBCL treatment remain unresolved, partly due to a limited understanding of biomarkers predicting response to rituximab or obinutuzumab.

One biomarker identified to predict decreased benefit from the addition of rituximab to fludarabine and cyclophosphamide in CLL treatment is the presence of *NOTCH1* mutation (Stilgenbauer et al., 2014). In contrast, obinutuzumab maintains beneficial effects in this CLL subgroup (Estenfelder et al., 2016). How the membrane-bound transcription factor NOTCH1 can interfere with rituximab-based chemoimmunotherapy is also unknown. NOTCH1 releases its intracellular domain (NICD1) after two cleavage steps executed by the disintegrin and metalloproteinases ADAM10 or ADAM17 and by the γ-secretase complex to up-regulate genes involved in B cell survival and resistance to apoptosis, proliferation, and differentiation (Borggrefe and Oswald, 2009; Fabbri et al., 2017; Santos et al., 2007). In B cell malignancies, most *NOTCH1* mutations result in a disruption of the PEST domain responsible for NICD1 inactivation and degradation (Fabbri et al., 2011; Weng et al., 2004; Stilgenbauer et al., 2014).

To characterize B-cell-intrinsic signaling events following rituximab and obinutuzumab treatment, we applied liquid chromatography-tandem mass spectrometry (LC-MS/MS)-based phosphoproteomics. We thereby uncovered an activation of pathways downstream of the BCR by rituximab as well as obinutuzumab treatment, identified differences between the two monoclonal antibodies, and discovered links between anti-CD20 treatment and NOTCH1 arising from an activation of the BCR signaling cascade.

## Results

### Activation of BCR signaling

**Figure 1:**
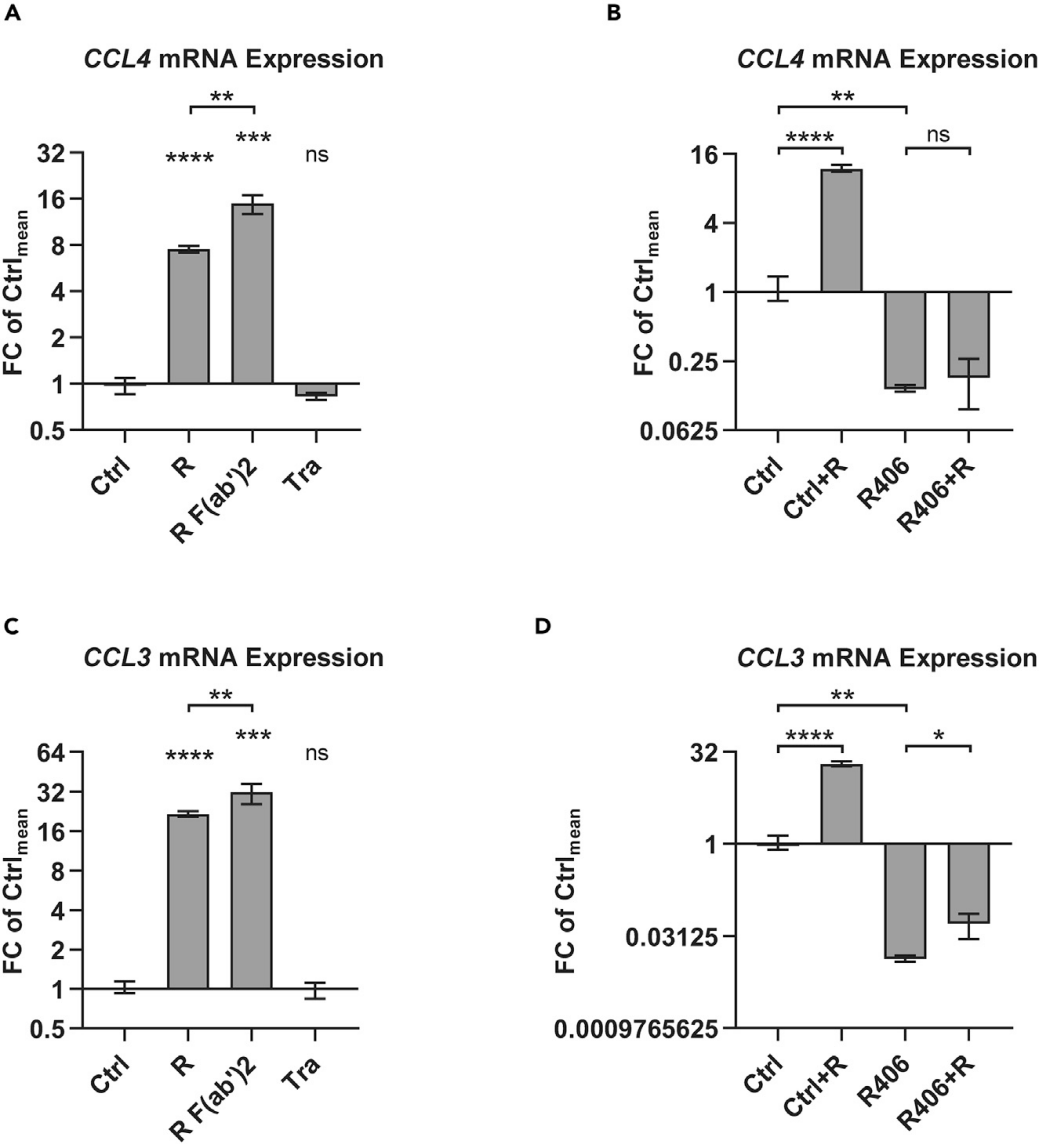
Rituximab activates B cell receptor signaling as inferred by *CCL4* and *CCL3* expression (A–D) *CCL4* (A + B) and *CCL3* (C + D) expression was assessed in SU-DHL4 cells by qRT-PCR after 150-min treatment with rituximab (R), rituximab F(ab‘)_2_ fragments (R F(ab‘)_2_), or with trastuzumab (Tra) relative to untreated control samples (Ctrl). Where applicable (B + D), cells were treated with the SYK inhibitor R406 or with DMSO vehicle control (Ctrl) for 48 h. Statistical significance was tested by unpaired parametric t tests based on 3 biological replicates for each treatment condition. Mean with range is plotted. ∗p<0.05, ∗∗p<0.01, ∗∗∗p<0.001, ∗∗∗∗p<0.0001, ns = not significant, as calculated by unpaired non-parametric t tests.

First, to validate a functionally relevant increase in BCR signaling by rituximab treatment, we measured *CCL4* and *CCL3* expression as established surrogates for BCR activation (Takahashi et al., 2015). Transcription of both genes was up-regulated following rituximab treatment (p < 0.0001). An increase in *CCL4* and *CCL3* expression was also observed after treating with rituximab F(ab’)_2_ fragments (p < 0.001), but not trastuzumab (Figure 1) demonstrating that the induction of BCR signaling was specific for CD20 binding and not engagement of the inhibitory FcγRIIB. R406 treatment to inhibit the spleen tyrosine kinase (SYK) reduced basal *CCL4* and *CCL3* expression levels (p < 0.01), diminished the increase in *CCL3* expression after rituximab treatment (mean fold changes 20.2 versus 3.8; p < 0.001), and completely abrogated *CCL4* up-regulation by rituximab (Figure 1), positioning signal generation by rituximab toward the proximal BCR signaling cascade. Increased BCR signaling as inferred by *CCL4* expression was also observed in MEC1 as well as in primary CLL cells after rituximab treatment (Figure S1).

**Figure 2:**
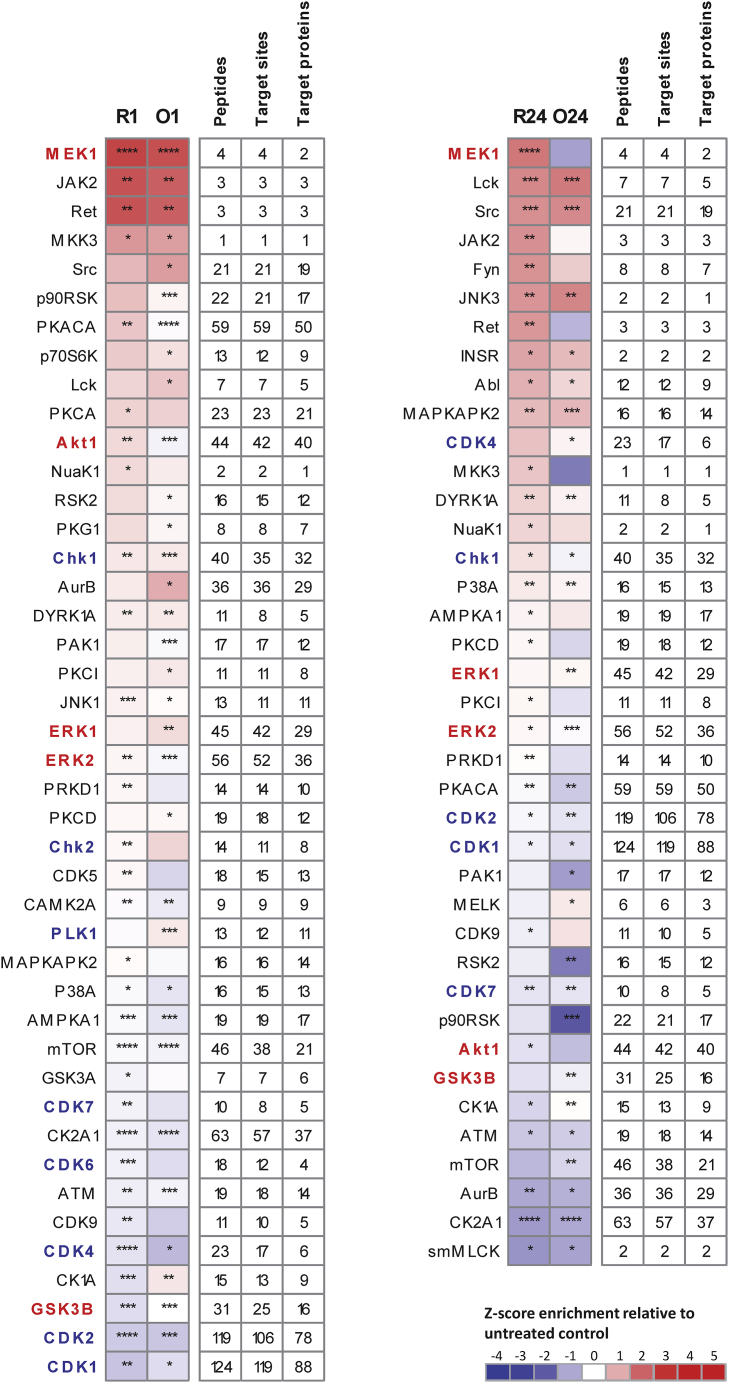
Kinase activity changes after rituximab or obinutuzumab treatment SU-DHL4 cells were treated with 5 μg/mL rituximab (R) or obinutuzumab (O) for 1 h (R1 and O1; left) or 24 h (R24 and O24; right), and changes in kinase activities were inferred relative to untreated controls. The heatmaps show the *Z* score enrichment of substrate groups for the different kinases calculated by the KSEA algorithm. Kinases belonging to the KEGG pathway “B-cell receptor signaling” are indicated in red, and those belonging to the KEGG pathway “cell cycle” are indicated in blue. Peptides: number of peptides containing a phosphorylation site regulated by respective kinase; Target sites: number of phosphorylation sites measured for respective kinase; Target proteins: number of proteins that the phosphorylation sites regulated by the respective kinase map to. ∗p<0.05, ∗∗p<0.01, ∗∗∗p<0.001, ∗∗∗∗p<0.0001 as inferred by the hypergeometric test followed by Benjamini-Hochberg multiple testing correction.

To refine our understanding of rituximab-induced signaling events within the BCR signaling cascade and compare them with signals generated by obinutuzumab treatment, we used LC-MS/MS-based phosphoproteomics to analyze SU-DHL4 lymphoma cells after treating with rituximab or obinutuzumab for 1 or 24 h. Considering the two time points in both treatment arms relative to untreated control samples, we identified 41 protein kinases after rituximab and 40 protein kinases after obinutuzumab treatment with significantly altered activity as inferred by KSEA. Thirty-two of these kinases were affected by both rituximab and obinutuzumab, suggesting a high concordance between the signaling pathways modified by both antibodies (Figure 2). Pathway enrichment analyses of the affected kinases revealed activation of pathways belonging to the BCR signaling cascade and down-regulation of cell cycle progression subsequent to both antibody treatments (Table S1).

Excessively strong signals from the BCR lead to autoimmune checkpoint activation, cell-cycle arrest, and B cell apoptosis as a physiologic mechanism to negatively select B cells with a specificity for autoantigens (Muschen, 2018). To explore the hypothesis that rituximab and obinutuzumab hijack this mechanism to elicit direct B cell killing, we next analyzed ERK, SYK, and the PI3K in more detail, as strong activation of these three kinases has been shown to drive B cell selection (Muschen, 2018).

### Differences between rituximab and obinutuzumab

**Figure 3:**
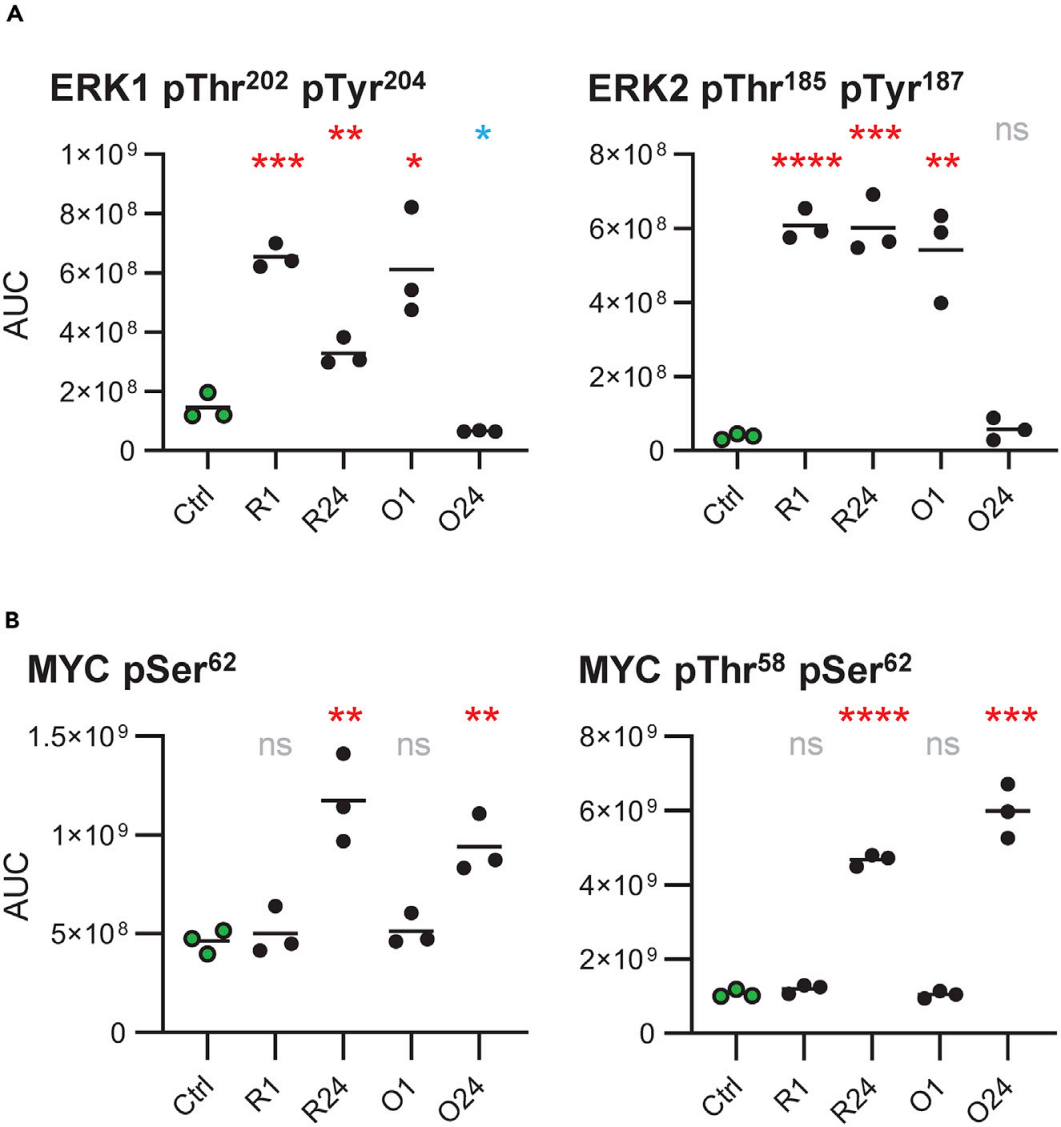
Rituximab and obinutuzumab induce ERK and MYC phosphorylation capable to induce B cell death (A) Calculated areas under the curve (AUC) for phosphopeptide ions dually phosphorylated on ERK1 Thr^202^/Tyr^204^ (left) and on ERK2 Thr^185^/Tyr^187^ (right) after treatment with rituximab (R) or obinutuzumab (O) for 1 or 24 h. Each of the three plotted biological replicates depict the average of the two analytical replicates. Phosphorylation changes were tested for statistical significance by unpaired non-parametric t tests calculated toward untreated control samples (Ctrl). Significant phosphorylation is indicated in red; significant de-phosphorylation is indicated in blue. (B) Calculated AUCs for phosphopeptide ions phosphorylated on MYC Ser^62^ (left) and dually phosphorylated on MYC Thr^58^/Ser^62^ (right). Line indicates mean. ∗p<0.05, ∗∗p<0.01, ∗∗∗p<0.001, ∗∗∗∗p<0.0001, ns = not significant, as calculated by unpaired non-parametric t tests.

The kinase with the strongest increase in activity at the 1 h time point after antibody treatment was identified as MEK, responsible for ERK activation (Figure 2). Consistently, ERK1 Thr^202^/Tyr^204^ and ERK2 Thr^185^/Tyr^187^ in the kinase activation loops had highly increased phosphorylation levels at this time point (4-fold and >10-fold increase, respectively; Figure 3A). The 24 h time point revealed lasting activity in the MEK-ERK signaling axis after rituximab treatment, whereas this longevity was not observed for obinutuzumab. The ERK target site MYC Ser^62^ had a 2-fold increase in phosphorylation at the 24 h time point uncovering activation of the MYC transcription factor. However, at the same time point, we also observed increased levels of Thr^58^/Ser^62^ doubly phosphorylated MYC (4- to 5-fold) representing an already de-activated form of the short-lived transcription factor (Figure 3B). In the absence of pro-survival stimuli, strong MYC activation can re-enforce apoptosis induction (Hoffman and Liebermann, 2008).

**Figure 4:**
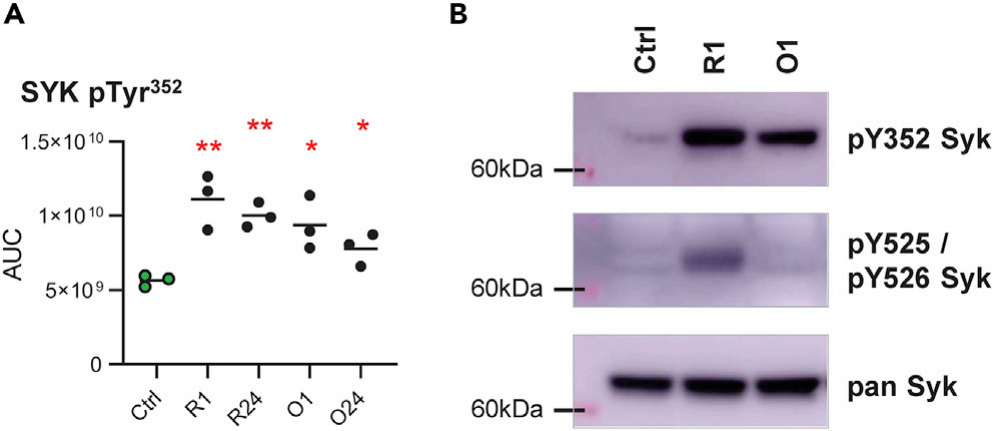
Obinutuzumab exceeds rituximab in supporting apoptosis induction by means of aberrant SYK phosphorylation. (A) Calculated AUCs for phosphopeptide ions containing the phosphorylation site Tyr^352^ on SYK. Line indicates mean. ∗p<0.05, ∗∗p<0.01, as calculated by unpaired non-parametric t tests. (B) Immunoblot detection of phospho-SYK Tyr^352^ and phospho-SYK Tyr^525^/Tyr^526^ in SU-DHL4 cells treated with 2.5 μg/ml rituximab (R) or obinutuzumab (O) for 1 h relative to untreated control samples (Ctrl; shown are representative results from one of four experiments).

SYK kinase activity was not significantly altered, although rituximab or obinutuzumab treatment increased the phosphorylation level of 11 and 9 SYK residues, respectively. The phosphorylation sites included SYK Tyr^352^ but not SYK Tyr^525/526^ located in the kinase activation loop (Figures 4A and S2). This constellation of phosphorylated Syk residues has previously been associated with B cell apoptosis (Muschen, 2018). We confirmed a lack of SYK Tyr^525/526^ phosphorylation after obinutuzumab treatment by immunoblotting, but revealed low levels of SYK Tyr^525/526^ phosphorylation by rituximab (Figure 4B).

**Figure 5:**
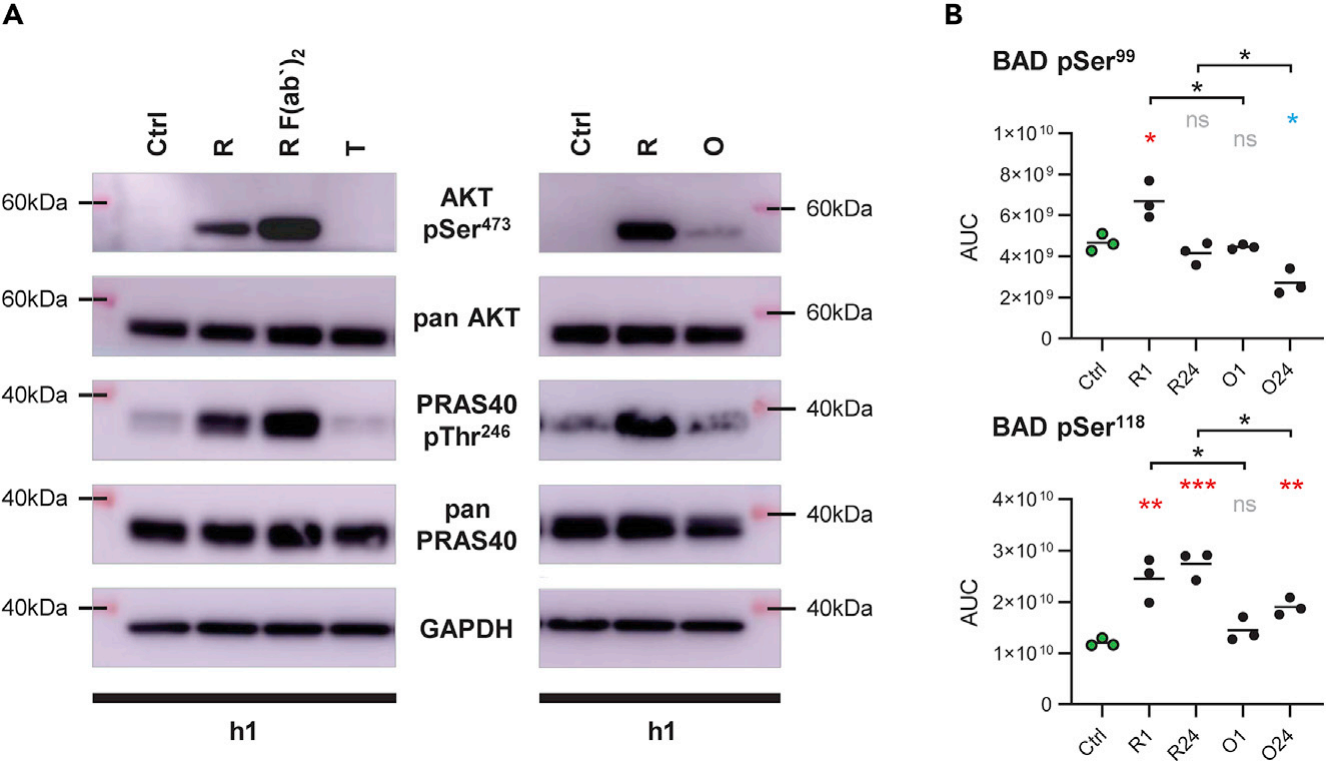
Rituximab more than obinutuzumab induces pro-survival signals (A) Immunoblot detection of phospho-AKT Ser^473^ and phospho-PRAS40 Thr^246^ in SU-DHL4 cells treated with 2.5 μg/ml rituximab (R), rituximab F(ab‘)_2_ fragments (R F(ab‘)_2_), trastuzumab (T), or obinutuzumab (O) for 1 h relative to untreated control samples (Ctrl). Shown are representative results from one of four experiments. (B) Calculated AUCs for phosphopeptide ions containing the phosphorylation site Ser^99^ (top) or Ser^118^ (bottom) on BAD. Line indicates mean. ∗p<0.05, ∗∗p<0.01, ∗∗∗p<0.001, ns = not significant, as calculated by unpaired non-parametric t tests.

Activation of the lipid kinase phosphatidylinositol 3-kinase (PI3K) was inferred by changes in the phosphorylation level of PI3K binding sites on CD19 and BCAP and by activity changes of downstream PI3K effectors (Werner et al., 2010). Increased tyrosine phosphorylation levels on CD19 and BCAP implied PI3K activation by rituximab as well as obinutuzumab, whereby more pronounced phosphorylation on CD19 Tyr^500^ after rituximab was suggestive of stronger PI3K activation following this treatment (Figure S3). However, of the three PI3K effectors MTOR, PDK1, and AKT, only AKT was found to be more active at the 1 h time point after rituximab treatment (Figure 2), likely resulting from direct AKT activation by Ca^2+^ flux following treatment with type I but not type II anti-CD20 monoclonal antibodies (Walshe et al., 2008; Yano et al., 1998). To validate AKT activation we assessed phosphorylation of AKT Thr^308^ and Ser^473^ by immunoblotting due to lack of evidence in our LC-MS/MS data. Although AKT Thr^308^ phosphorylation was not observed (data not shown), we found a strong increase in AKT Ser^473^ phosphorylation at 1 h after rituximab and rituximab F(ab’)_2_ treatment with fading signals at the 24 h time point (Figure S4). This result corresponded with phosphorylation of the AKT target site PRAS40 Thr^246^. Only low levels of phospho-AKT Ser^473^ were detected after obinutuzumab treatment and no increase in AKT Ser^473^ phosphorylation was observed after isotype control trastuzumab treatment (Figure 5A).

Because of the known powerful pro-survival effects of AKT (Werner et al., 2010), the differing ability of rituximab and obinutuzumab to activate this kinase constituted a decisive difference between the two monoclonal antibodies at the molecular level. Consistently, BAD Ser^99^, as an important AKT target site with key role in inhibition of apoptosis, was found phosphorylated only after rituximab treatment (p = 0.024; 1 h). In addition, rituximab more than obinutuzumab increased levels of Ser^118^ phosphorylation on BAD (p = 0.0074 at 1 h; p = 0.0007 at 24 h; Figure 5B). Phosphorylation on Ser^99^ and Ser^118^ sequesters pro-apoptotic BAD in the cytosol and impairs its inhibitory effects on anti-apoptotic BCL2 and BCL-xL so that rituximab more than obinutuzumab may diminish direct B cell death by reinforcing BCL2-mediated anti-apoptotic signals (Masters et al., 2001; Zha et al., 1996). In line with this notion, SU-DHL4 cell viability was more strongly decreased by obinutuzumab than rituximab treatment (Alduaij et al., 2011; Herter et al., 2013).

### Links between BCR and NOTCH1 signaling

**Figure 6:**
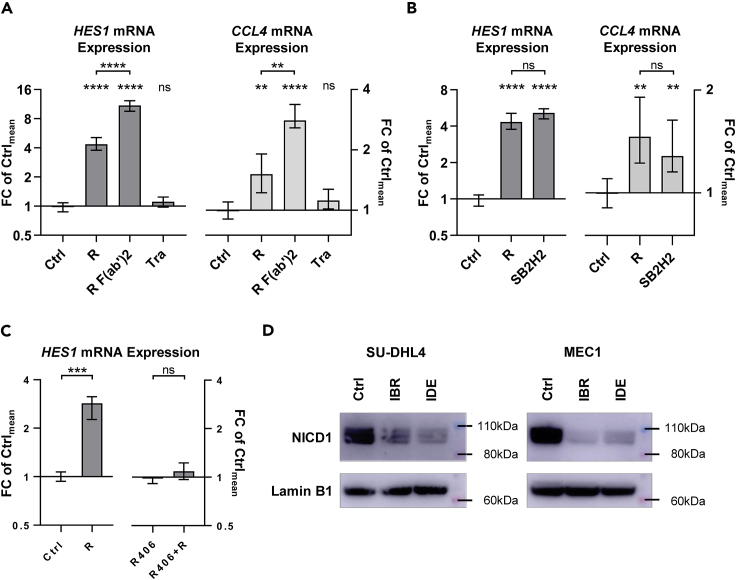
NOTCH1 and BCR signaling are synergized (A) *HES1* (left) and *CCL4* (right) expression was assessed in SU-DHL4 cells by qRT-PCR after 150 min treatment with 2.5 μg/ml rituximab (R), rituximab F(ab‘)2 fragments (R F(ab’)_2_), or trastuzumab (T) relative to untreated control samples (Ctrl). Statistical significance was tested by unpaired non-parametric t tests based on 8 biological replicates for the control samples and 4 biological replicates for each treatment condition. (B) *HES1* and *CCL4* expression in SU-DHL4 cells after 150 min of treatment with 2.5 μg/ml rituximab (R) or SB2H2 relative to untreated control samples (Ctrl). (C) *HES1* expression in SU-DHL4 cells pre-treated with vehicle control (Ctrl, left) or R406 (5 μM; right) before treatment with 2.5 μg/mL rituximab (R) for 150 min. (D) Immunoblot detection of nuclear NICD1 protein levels in SU-DHL4 cells (left) and MEC1 cells (right) after 48 h treatment with ibrutinib (IBR; 1 μM) or idelalisib (IDE; 5 μM) relative to samples treated with vehicle control (Ctrl). Shown are representative results for one of four experiments on SU-DHL4 cells and one of two experiments on MEC1 cells. Mean with range is plotted. ∗p<0.05, ∗∗p<0.01, ∗∗∗p<0.001, ∗∗∗∗p<0.0001, ns = not significant, as calculated by unpaired non-parametric t tests.

We next sought to understand the link between rituximab treatment and NOTCH1 signaling. Based on publications showing increased ADAM activity upon Ca^2+^ flux and PI3K/MAPK-dependent phosphorylation changes (Herzog et al., 2014; Fan et al., 2003; Zhang et al., 2006; Li et al., 2018), we hypothesized that anti-CD20 monoclonal antibodies could enhance ADAM10/ADAM17-mediated NOTCH1 cleavage. To quantify short-term rises of NOTCH1 signaling, we assessed expression changes of its target gene *HES1* by qRT-PCR. Results obtained for rituximab and its control antibodies in SU-DHL4 cells correlated with BCR activation after rituximab treatment, as demonstrated by *CCL4* expression (Figure 6A). An increase in *HES1* expression after rituximab treatment was also validated in primary CLL cells (Figure S5).

The increases in *HES1* expression observed after rituximab treatment in SU-DHL4 cells matched those induced by SB2H2 treatment, which cross-links and activates the IgG BCR of SU-DHL4 directly (Figure 6B). Abolishment of Ca^2+^ flux and BCR signaling by R406 completely abrogated the increase in *HES1* and *CCL4* expression after rituximab exposure (Figure 6C). Treatment with the PI3K inhibitor idelalisib and the BTK inhibitor ibrutinib clearly reduced nuclear NICD1 protein levels without obvious difference between both drugs (Figure 6D), but did not prevent the increase in *HES1* expression after rituximab treatment (Figure S6).

**Figure 7:**
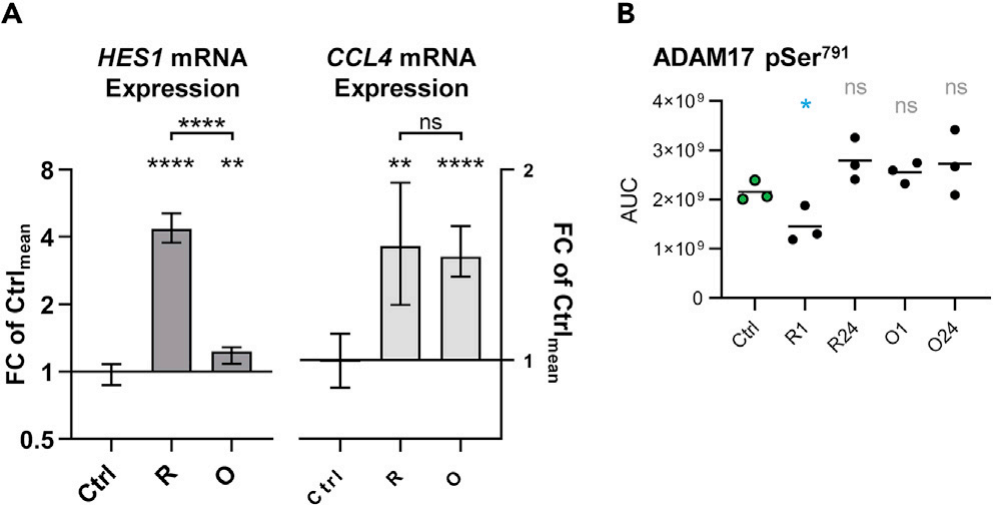
Rituximab induces NOTCH1 signaling more strongly than obinutuzumab (A) *HES1* (left) and *CCL4* (right) expression in SU-DHL4 cells after 150 min of treatment with 2.5 μg/ml rituximab (R) or obinutuzumab (O) relative to untreated controls (Ctrl). Mean with range is plotted. (B) Calculated AUCs for phosphopeptide ions containing the phosphorylation site Ser^791^ on ADAM17. Line indicates mean. ∗p<0.05, ∗∗p<0.01, ∗∗∗p<0.001, ∗∗∗∗p<0.0001ns = not significant, as calculated by unpaired non-parametric t tests.

Direct comparison of rituximab and obinutuzumab treatments in SU-DHL4 cells revealed a much subtler increase in *HES1* expression following obinutuzumab treatment despite a comparable increase in *CCL4* expression (Figure 7A). This result was consistent with a role for Ca^2+^ flux during NOTCH1 activation (Le Gall et al., 2009; Arruga et al., 2020). In addition, LC-MS/MS data revealed significant de-phosphorylation of ADAM17 Ser^791^ only after rituximab treatment (p=0.048; Figure 7B), which has been shown previously to enhance the activity of ADAM17 (Fan et al., 2003). The kinetics of ADAM17 Ser^791^ de-phosphorylation followed those observed in the PI3K/AKT pathway suggesting positive feedback from this pathway to the NOTCH1 receptor.

We finally validated NOTCH1 activation by rituximab at protein level. Western blot analysis for NICD1 showed increased NOTCH1 signaling after rituximab treatment in SU-DHL4 cells and three independent CLL cases (Figure S7). However, increased NOTCH1 signaling after treatment with rituximab F(ab’)_2_ fragments was inconsistently observed in primary CLL cells. This may be afforded by heterogeneous basal activity levels of the NOTCH1 receptor as well as in the B cell receptor signaling cascade. In contrast to results obtained with the SU-DHL4 cell line, trastuzumab treatment increased NOTCH1 cleavage in all three primary CLL samples. Trastuzumab binds to the Fc gamma receptor expressed on immune effector cells, and we hence reasoned that in addition to B cell-intrinsic modes of NOTCH1 activation, cleavage of NOTCH1 may, furthermore, be enhanced by effector cell activation. To address this hypothesis, we correlated the increase in *HES1* expression in eight independent CLL samples with the respective increase in *CCL2* expression (Figure S8). The latter was used as a surrogate marker for the presence of activated monocytes (Schulz et al., 2011). Our results supported a positive correlation between the extent of monocyte activation and NOTCH1 signaling strength (R2=0.79; p<0.0001), which may result from a recruitment of NOTCH1 ligand expressing monocytes to CLL cells and/or from an activation of B cell-intrinsic signaling pathways subsequent to a release of signaling molecules into the culture medium by activated effector cells (Lopez-Guerra et al., 2020).

### Discussion

Our results demonstrate that rituximab and obinutuzumab both hijack the BCR signaling cascade, but in different directions. Excessive ERK and MYC activation by riyuximab and obinutuzumab treatment supported the hypothesis that both anti-CD20 monoclonal antibodies induce direct cell killing via signals generated in the BCR signaling cascade (Muschen, 2018; Hoffman and Liebermann, 2008). However, as demonstrated by an aberrant SYK phosphorylation pattern, obinutuzumab more effectively shifted the balance of these signals toward death, whereas rituximab engaged stronger signals associated with survival, comprising pro-survival SYK Tyr^525/526^ phosphorylation, AKT activation, BAD Ser^99^ and Ser^118^ phosphorylation, and NOTCH1 activation. The stronger activation of NOTCH1 by rituximab acts in concert with AKT activation, because *BCL2* is a target gene of the NICD1 transcription factor and up-regulated *BAD* transcription has been observed in CLL with overactive NOTCH1 signaling (Fabbri et al., 2017). Hence, it appears that rituximab can reinforce BCL2-mediated apoptotic resistance at both the protein and gene expression levels thereby diminishing the degree of B cell killing (Werner et al., 2010; Fabbri et al., 2017). PEST-domain *NOTCH1* mutations therefore should reinforce pro-survival signals after rituximab treatment due to their activating effect on NICD1 potentially explaining the reduced benefit of adding rituximab to chemotherapy in *NOTCH1* mutant CLL (Stilgenbauer et al., 2014; Weng et al., 2004).

Clinical trial data suggest that our results could influence clinical practice. CLL and follicular lymphoma, which benefit from the use of obinutuzumab, are both characterized by high-level BCL2 expression (Goede et al., 2014, 2015; Marcus et al., 2017), whereas DLBCL is a more heterogeneous entity (Schmitz et al., 2018). Focusing on germinal center B cell-type DLBCL encompassing a considerable number of cases with genetic alterations affecting BCL2 family members revealed a trend toward PFS improvement using obinutuzumab (Vitolo et al., 2017; Schmitz et al., 2018). In contrast, PFS analysis within the activated B cell (ABC-)type DLBCL showed almost identical results for both treatment arms, which may be afforded by frequent co-occurrence of genetic aberrations affecting BCL2 family members with those affecting proximal BCR signaling (Vitolo et al., 2017; Schmitz et al., 2018). The latter have been associated with chronic active BCR signaling bringing about a lower capacity for rituximab and obinutuzumab to alter intrinsic B cell signals (Schmitz et al., 2018), hence likely reducing the molecular advantages observed for obinutuzumab. Considering these observations, our results warrant refined analyses of respective trial cohorts to identify biomarkers indicating where obinutuzumab should become standard of care in the treatment of B cell lymphoma.

BCR signaling is a driver of lymphomagenesis (Niemann and Wiestner, 2013) to the extent that its activation bears the risk of providing growth stimuli to lymphoma cells if the apoptotic threshold is not reached. This risk is higher using rituximab, which may be reflected in loss of CD20 expression at relapse post rituximab treatment if lymphoma cells fail to reach the threshold because of (ultra-)low CD20 expression (Hiraga et al., 2009; Tomita, 2016). Moreover, the increase in BCR signaling after anti-CD20 treatment interacts with the modes of action of concomitant drugs. Response to cell cycle-dependent cytostatic agents may be reduced by an arrest in the cell cycle, and targeting CD20 as well as BTK may lead to partial antagonization of each other's effects, providing a rationale for the lack of PFS improvement observed in CLL after adding rituximab to ibrutinib treatment (Burger et al., 2019). In contrast, PI3K and BCL2 inhibitors likely exert complementary effects on anti-CD20 therapy. Our results thus provide new explanatory approaches for therapy resistance in B cell lymphoma treatment and help to refine patient selection for rituximab or obinutuzumab and to improve drug sequencing within anti-CD20 monoclonal antibody containing treatment protocols.

Furthermore, we identify the membrane-bound transcription factor NOTCH1 as a connective link between the BCR signaling cascade and genes promoting B cell survival and proliferation. NOTCH1 cleavage upon Ca^2+^ flux allows rapid NICD1 release after BCR activation, and modulation of ADAM17 cleavage activity by (de-)phosphorylation allows an adjustment of NOTCH1 signaling strength. ADAM10 was shown to cleave NOTCH1 after ligand binding, whereas ADAM17 has been associated with ligand-independent NOTCH1 activation (Bozkulak and Weinmaster, 2009). Activation of ADAM17 by signals generated through an (auto-)active BCR may therefore explain high NICD1 protein levels observed in *NOTCH1* wild-type peripheral blood CLL cells that lack contact to NOTCH1 ligands (Fabbri et al., 2017). Moreover, our data suggest that NOTCH1 cleavage in B cells is also dependent on the level of immune effector cell activation in the microenvironment allowing an adaption of NOTCH1 signaling in B cells to the degree of inflammation. Taken together, our results warrant more detailed studies aiming at a better understanding of ADAM10/17 regulation in B cells to exploit the underlying mechanisms for effective suppression of NOTCH1 signaling.

In conclusion, our results demonstrate unexpected complexity by which rituximab and obinutuzumab interfere with signaling pathways essential for B cell lymphoma pathogenesis and treatment. This new insight provides impetus to better personalize the choice of rituximab or obinutuzumab for anti-CD20 treatment, to optimize the design of protocols encompassing anti-CD20 monoclonal antibodies, and to develop new strategies for the treatment of NOTCH1-driven B cell lymphoma.

### Limitations of study

A limitation of the study is that B cell-intrinsic signaling processes after anti-CD20 treatment have been determined in one cell line only and that the results obtained therefore lack evidence for general applicability. The work is hypothesis generating and paves the way toward subsequent studies addressing each conclusion drawn.

### Resource availability

#### Lead contact

Jennifer Edelmann, MD, PhD.

Department of Internal Medicine III, Ulm University, Albert-Einstein-Allee 23, 89,081 Ulm, Germany.

Mail: jennifer.edelmann@uni-ulm.de.

Tel: +49 (0)731 500 45849.

#### Materials availability

This study did not generate new unique reagents.

#### Data and code availability

The mass spectrometry proteomics data generated during this study have been deposited to the ProteomeXchange Consortium via the PRIDE partner repository with the dataset identifier PXD023572 and 10.6019/PXD023572.

## Methods

All methods can be found in the accompanying Transparent Methods supplemental file.

## Supplemental information

Document S1. Transparent methods and Figures S1–S8

Table S1. Pathways significantly affected by changes in protein kinase activities, related to Figure 2
