# Supplementary material for: Rituximab and obinutuzumab differentially hijack the B cell receptor and NOTCH1 signaling pathways
Source: iScience. 2021 Jan 22;24(2):102089. doi: 10.1016/j.isci.2021.102089 (PMC7878992; doi:10.1016/j.isci.2021.102089)
Supplement: Document S1. Transparent methods and Figures S1–S8 [file mmc1.pdf]

iScience, Volume *24*

Supplemental Information

Rituximab and obinutuzumab differentially hijack the B cell receptor and NOTCH1 signaling pathways

Jennifer Edelmann, Arran D. Dokal, Emma Vilventhraraja, Karlheinz Holzmann, David Britton, Tetyana Klymenko, Hartmut Döhner, Mark Cragg, Andrejs Braun, Pedro Cutillas, and John G. Gribben

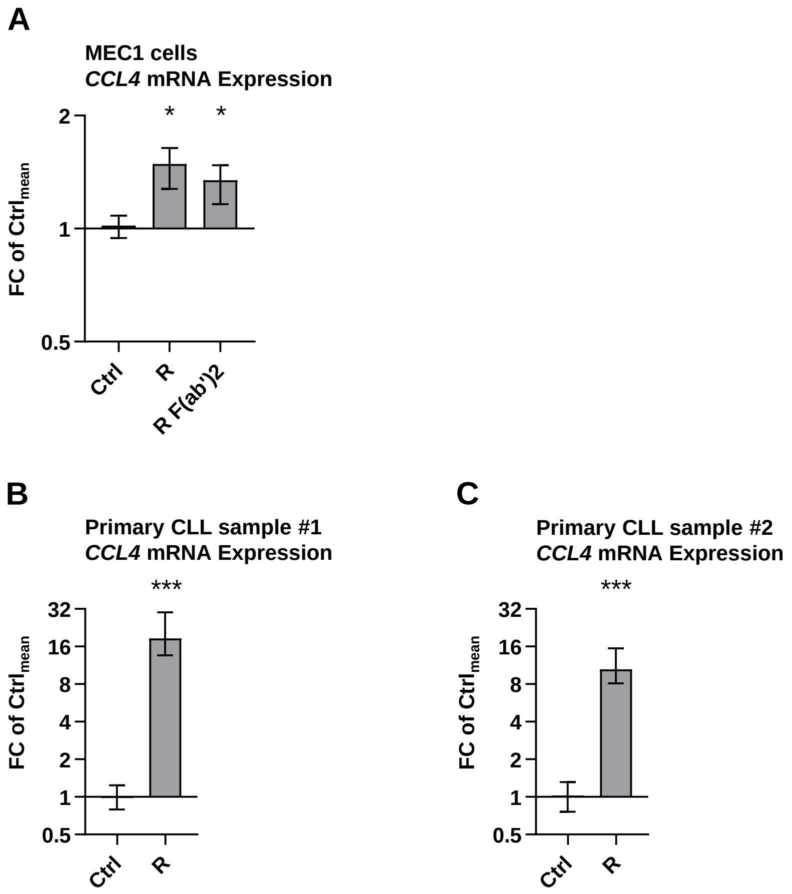

Supplementary Figure S1:

Validation of activated B-cell receptor signaling by rituximab treatment in MEC1 cells and two primary CLL samples.

Related to Figure 1.

CCL4 expression was assessed in MEC1 cells (**A**) and in primary CLL cells (**B** + **C**) after 150 minutes of treatment with rituximab (R) or rituximab $F(ab')_2$ fragments (R $F(ab')_2$) relative to untreated control samples (Ctrl). Statistical significance was tested by unpaired parametric t-tests based on 3 biological replicates for each treatment condition in the case of MEC1 cells and based on 5 biological replicates in the case of primary CLL samples. Mean with range is plotted. *<0.05, **<0.01, ***<0.001; as calculated by unpaired non-parametric t-tests.

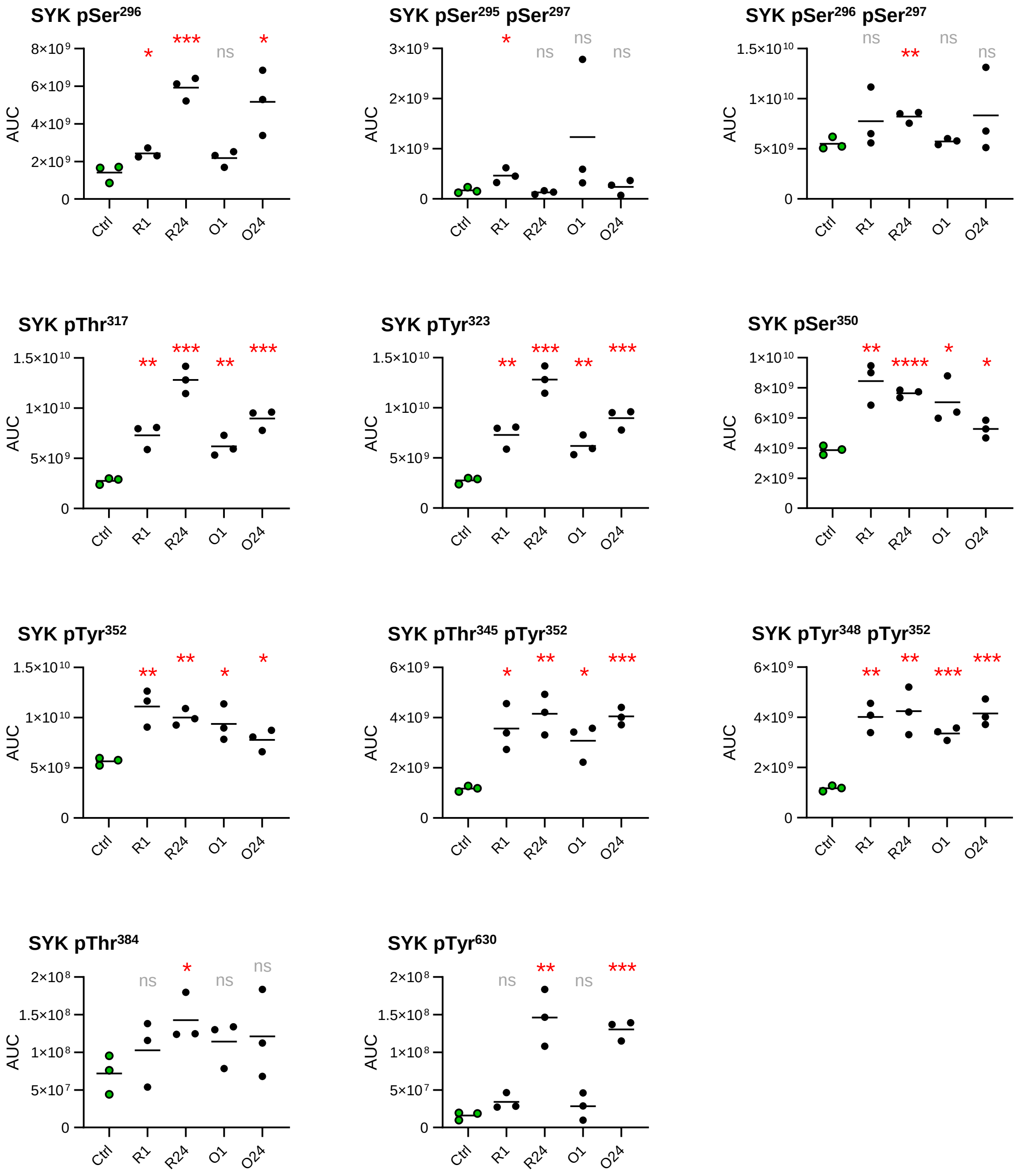

Supplementary Figure S2:

SYK phosphorylation after rituximab or obinutuzumab treatment.

Related to Figure 4.

Calculated AUCs for phosphopeptide ions containing the phosphorylation sites indicated above on SYK. Line indicates mean. *<0.05, **<0.01, ***<0.001, ns = not significant; as calculated by unpaired non-parametric t-tests.

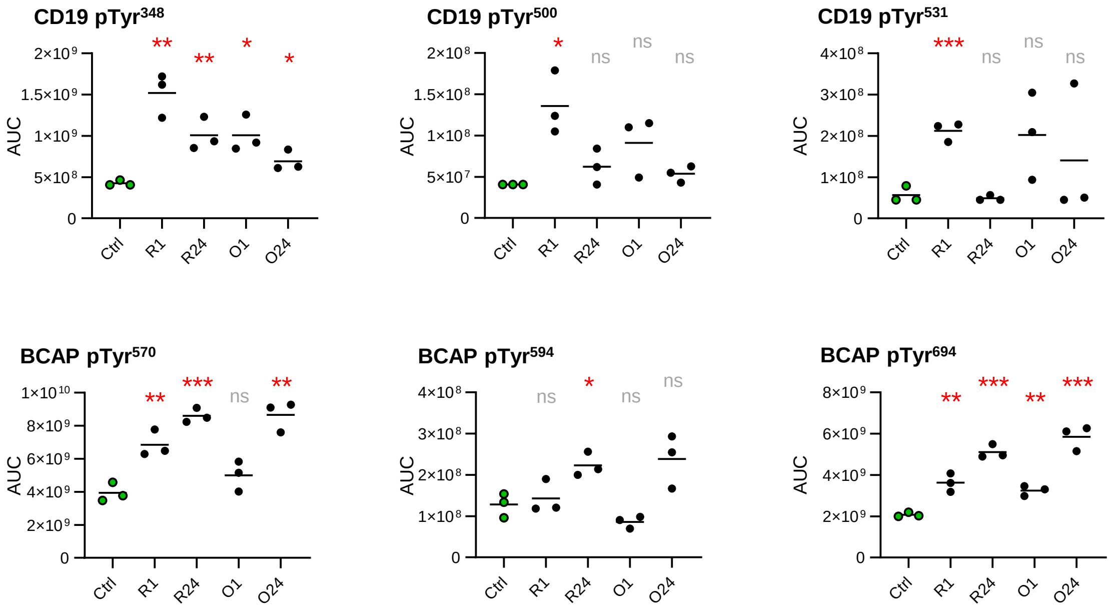

Supplementary Figure S3:

CD19 and BCAP phosphorylation after rituximab or obinutuzumab treatment.

Related to Figure 5.

Calculated AUCs for phosphopeptide ions containing the phosphorylation sites indicated above on CD19 (**top**) or BCAP (**bottom**). Line indicates mean. *<0.05, **<0.01, ***<0.001, ns = not significant; as calculated by unpaired non-parametric t-tests.

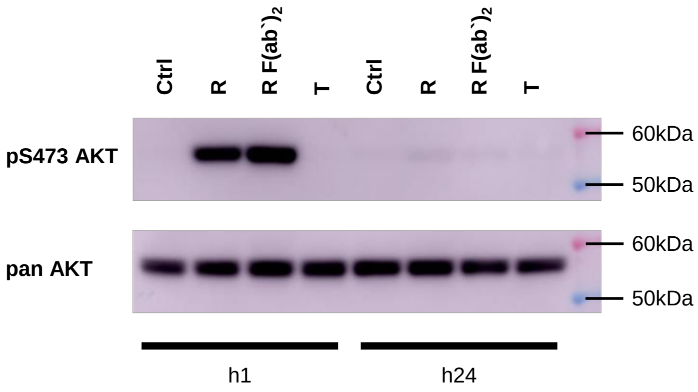

Supplementary Figure S4:

AKT activation was restricted to early time-points after rituximab treatment.

Related to Figure 5.

Immunoblot detection of phospho-AKT Ser^{473} in SU-DHL4 cells treated with rituximab (R), rituximab $F(ab')_2$ fragments (R $F(ab')_2$), or trastuzumab (T) for one hour (**left**) or 24 hours (**right**) relative to untreated control samples (Ctrl).

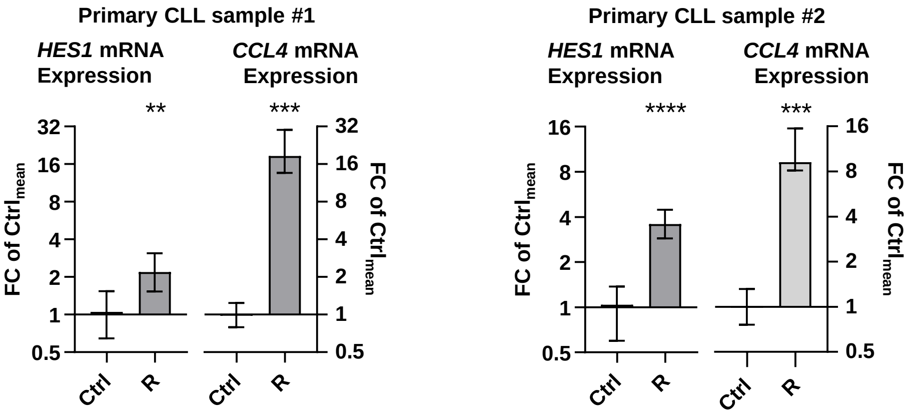

Supplementary Figure S5:

Validation of activated NOTCH1 signaling by rituximab treatment in two primary CLL samples.

Related to Figure 6.

HES1 and *CCL4* expression was assessed in primary CLL cells after 150 minutes of treatment with rituximab (R) relative to untreated control samples (Ctrl). Statistical significance was tested by unpaired parametric t-tests based on 5 biological replicates for each treatment condition. Mean with range is plotted. *<0.05, **<0.01, ***<0.001; as calculated by unpaired non-parametric t-tests.

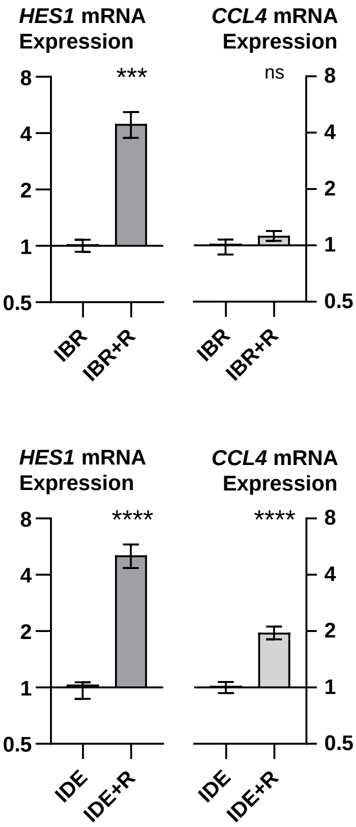

Supplementary Figure S6:

Treatment with ibrutinib or idelalisib did not prevent the increase in NOTCH1 signaling after rituximab treatment.

Related to Figure 6.

HES1 (**left**) and *CCL4* (**right**) expression was assessed in SU-DHL4 cells by qRT-PCR after 150 min of treatment with rituximab (R) relative to untreated control samples (Ctrl). Cells were pre-treated with the BTK inhibitor ibrutinib (**top**) or with the Pi3K inhibitor idelalisib (**bottom**) for 48 hours. Statistical significance was tested by unpaired parametric t-tests based on 4 biological replicates for each treatment condition. Mean with range is plotted. *<0.05, **<0.01, ***<0.001, ns = not significant; as calculated by unpaired non-parametric t-tests.

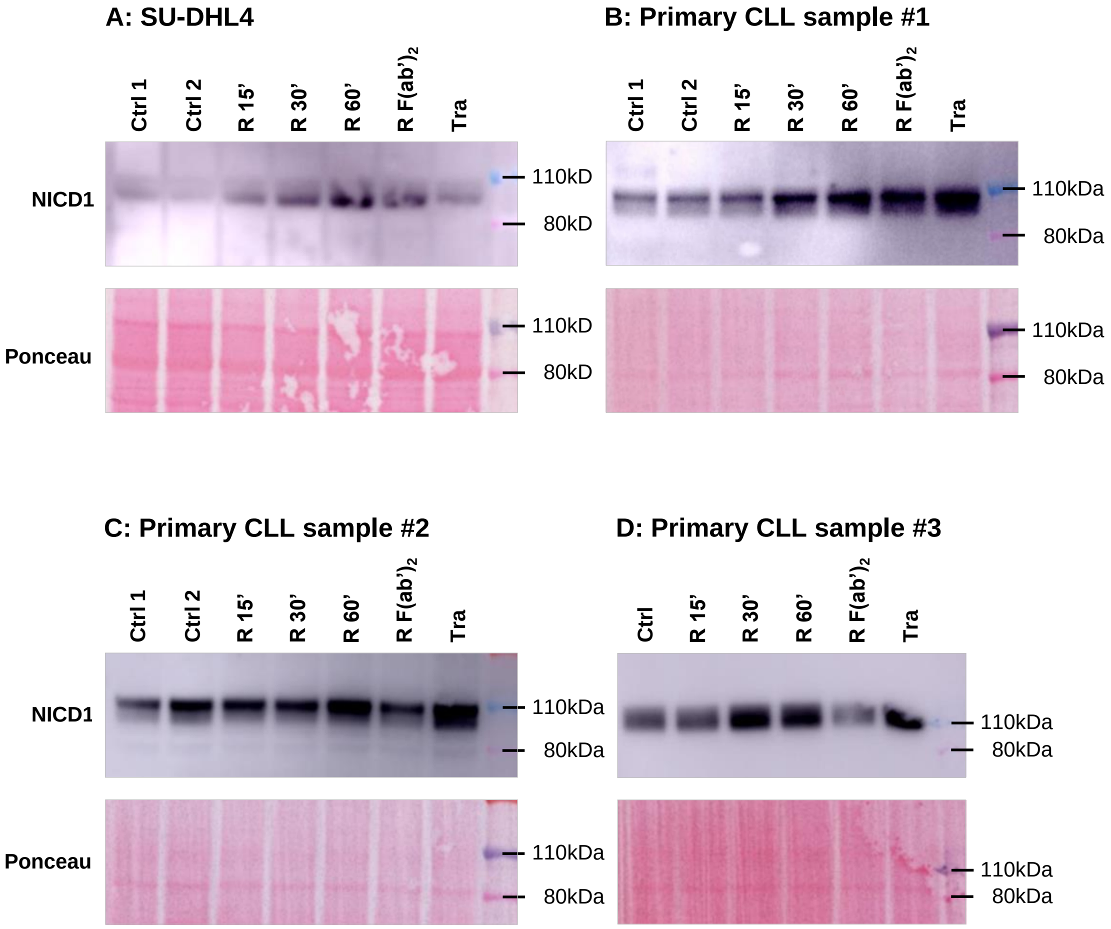

Supplementary Figure S7:

In primary CLL cells rituximab can increase NOTCH1 signaling via its F(ab')2 fragments and via its Fc-fragment.

Related to Figure 6.

Immunoblot detection of the NOTCH1 intracellular domain (NICD1) in SU-DHL4 cells (**A**) and in three primary CLL samples (**B-D**) treated with rituximab (R) for 15, 30 and 60 minutes, rituximab F(ab')$_2$ fragments (R F(ab')$_{2;}$) for 60 minutes, or trastuzumab (Tra) for 60 minutes relative to untreated control samples (Ctrl).

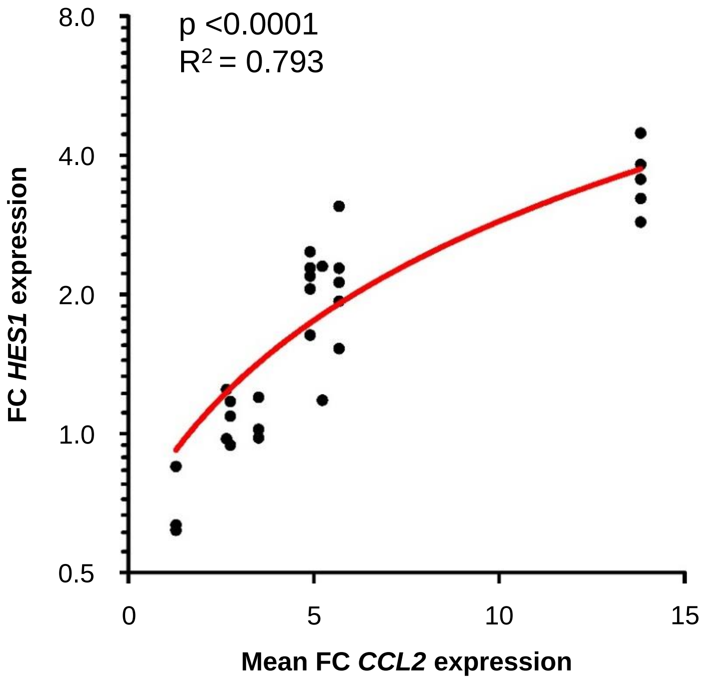

Supplementary Figure S8:

The increase in *HES1* expression after rituximab treatment correlated with the mean fold change of *CCL2* expression, used as surrogate marker for monocyte activation.

Related to Figure 6.

HES1 and *CCL2* expression was assessed in 8 individual CLL samples by qRT-PCR after 150 min of treatment with rituximab relative to untreated controls. Two to five biological replicates were used for each sample and treatment condition. The fold change (FC) of *HES1* expression was plotted against the mean FC of *CCL2* expression. Linear regression analysis was used to test for an association between the increase in *HES1* and *CCL2* expression.

Transparent Methods

Cells lines and patient samples

The B-cell lines SU-DHL4 derived from a germinal center B-cell type DLBCL (Epstein and Kaplan, 1979) and MEC1 derived from CLL in prolymphocytoid transformation (Stacchini et al., 1999) were obtained from American Type Culture Collection (ATCC, Manassas, VA, USA).

Peripheral blood samples were obtained from CLL patients attending St. Bartholomew's Hospital (Barts) and consenting to use of specimens for research. Peripheral blood mononuclear cells (PBMCs) were isolated via density gradient centrifugation (Lymphoprep®, Stemcell Technologies®, Vancouver, Canada) and enriched for B-cells via immunomagnetic beads against CD19 (MACS®, Miltenyi Biotec®, Bergisch Gladbach, Germany). To prevent activation of the NOTCH1 receptor during the isolation procedure, contact of cells with EDTA was avoided by the use of heparin monovettes and preparation of EDTA-free MACS sorting buffer. After sorting, CLL cells were used for downstream experiments immediately. White blood cell counts for the CLL samples used for Western blot analysis are provided below (as measured on the day of sample acquisition):

CLL1 WBC: 158.0 x 10^9/l

CLL2 WBC: 409,4 x 10^9/l

CLL3 WBC: 73.8 x 10^9/l

Reagents

Cells were treated with the two anti-CD20 monoclonal antibodies rituximab and obinutuzumab. Trastuzumab was used as an isotype control. F(ab')$_2$ fragments were used to

study Fc-independent effects resulting exclusively from CD20 binding. The IgG B-cell receptor of SU-DHL4 was cross-linked by SB2H2.

Rituximab, trastuzumab and obinutuzumab were obtained from the local pharmacy of St. Bartholomew's hospital, London, United Kingdom. SB2H2 and rituximab $F(ab')_2$ fragments were in-house productions at the Centre of Cancer Immunology at Southampton University, Prof. Mark Cragg, and received as gifts. Hybridoma cell lines secreting the respective monoclonal antibodies were cultured and secreted antibodies were purified from the culture supernatant using protein A columns (GE Healthcare, Chicago, IL, USA). Purity of in-house monoclonal antibodies was assessed by electrophoresis (Beckman EP system, Beckman Coulter, Pasadena, CA, USA). Rituximab $F(ab')_2$ fragments were produced by standard pepsin digestions. The kinase inhibitors R406, ibrutinib and idelalisib were purchased from Selleckchem® at a concentration of 10mM/1ml in DMSO (Houston, TX, USA).

Cell culture

The SU-DHL4 cell line was maintained in Roswell Park Memorial Institute (RPMI) 1640 medium (Sigma-Aldrich®); the MEC1 cell line in Dulbecco's Modified Eagle Medium (DMEM; Sigma-Aldrich®). Medium was supplemented with 10% fetal bovine serum (FBS; Life Technologies®, Carlsbad, CA, USA) and 1% Penicillin/Streptomycin (Sigma-Aldrich®). Both cell lines were maintained at a concentration of 0.7 x 10^6 cells/ml at 37 °C and 5% CO2.

Monoclonal antibody treatment of SU-DHL4 cells and primary CLL cells

To assess signaling changes in the B-cell receptor cascade after monoclonal antibody treatment (*CCL4* and *CCL3* expression; phosphorylation of SYK, AKT and PRAS40) cells were re-suspended in their cell culture medium at a concentration of 1 x 10^6 cells/ml and treated with

the respective antibody at a concentration of 5 µg/ml for 1 h, 2.5 h or 24 h. Whole cell lysates for protein analysis were obtained after 1 h or 24 h. RNA was isolated after 2.5 h.

To assess short-term changes in NOTCH1 signaling after monoclonal antibody treatment (*HES1* alongside *CCL4* expression), SU-DHL4 cells were re-suspended in PBS at a concentration of 5 x 10^6 cells/ml. Immediately after re-suspension, cells were treated with the respective monoclonal antibody at a concentration of 2.5 µg/ml and kept in the incubator at 37 °C and 5% CO2 for 1 h. After 1 h, two volumes of RPMI were added to one volume of PBS and cells were kept in the incubator for another 1.5 hours before RNA isolation.

To assess long-term changes in NOTCH1 signaling, SU-DHL4 and MEC1 cells were used directly from the culture medium supplemented with inhibitors or vehicle control for 48 h (see below). Nuclear cell lysates were used for NICD1 immunoblots.

Treatment of SU-DHL4 and MEC1 cells with kinase inhibitors

The kinases Syk, Btk and Pi3K were inhibited by R406, ibrutinib and idelalisib, respectively. Cells were exposed to the inhibitors for 48 hours before subsequent experiments were conducted. Inhibitors were used at the following concentrations:

R406 and idelalisib	5 µM
Ibrutinib	1 µM

DMSO vehicle controls were kept alongside. PBS and culture medium added during the course of an experiment were supplemented with the respective inhibitor at concentrations mentioned above or with vehicle control.

Cell viability in each inhibitor and control condition was >90% after 48 hours.

Protein immunoblotting

For SYK, AKT and PRAS40 immunoblotting from whole cell lysates, cells were lysed with the Qproteome Mammalian Protein Prep Kit (Qiagen®, Hilden, Germany) according to the manufacturer's protocol. For NICD1 immunoblotting from nuclear protein fractions, cells were processed with NE-PER Nuclear and Cytoplasmic Extraction Reagents (Thermo Scientific, Waltham, MA, USA). Protein concentrations were determined by averaging three to four technical replicates measured on the NanoDrop® ND-1000 by using the Bradford dye-binding method (Protein Assay Dye Reagent Concentrate; Bio-Rad Laboratories, Hercules, CA, USA).

Lysates were separated by NuPAGE 4-12% Bis-Tris gels (Thermo Fisher Scientific) and transferred to PVDF membranes (Immobilon®, 0.45 µm pore size; Burlington, MA, USA) by wet electroblotting (17 hours, 30 Volts). Membranes were blocked with TBS with 1% skim milk powder (Sigma; St. Louis, MO, USA) and incubated with primary antibody for 1 hour at room temperature (see list of antibodies below). Following incubation, each membrane was washed four times with TBS supplemented with 0.1% Tween® 20 (Sigma-Aldrich, St. Louis, MO, USA), before incubation with a secondary horseradish peroxidase conjugated goat anti-rabbit antibody (GE Healthcare). Protein bands were visualized by using ECL Prime Western Blotting Detection Reagent (GE Healthcare) and the Amersham 600 imager (GE Healthcare).

The following primary antibodies were used for western blot analysis:

Cleaved NOTCH1 (Val1744)(D3B8) Rabbit mAb	Cell Signaling Technology®
Syk (D3Z1E) XP® Rabbit mAb	Cell Signaling Technology®
Phospho-Zap-70 (Tyr319)/Syk (Tyr352) (65E4) Rabbit mAb	Cell Signaling Technology®
Phospho-Syk (Tyr525/526) (C87C1) Rabbit mAb	Cell Signaling Technology®
Akt (pan) (C67E7) Rabbit mAb	Cell Signaling Technology®
Phospho-Akt (Ser473) (D9E) XP® Rabbit mAb	Cell Signaling Technology®
Phospho-Akt (Thr308) (D25E6) XP® Rabbit mAb	Cell Signaling Technology®

PRAS40 (D23C7) XP® Rabbit mAb	Cell Signaling Technology®
Phospho-PRAS40 (Thr246) (C77D7) Rabbit mAb	Cell Signaling Technology®
GAPDH (D16H11) XP® Rabbit mAb	Cell Signaling Technology®
Anti-Lamin B1 antibody (ab16048) Rabbit polyclonal antibody	Abcam®

Quantitative real-time polymerase chain reaction (qRT-PCR)

RNA was isolated by using the RNeasy Mini Kit (Qiagen®). Complementary DNA (cDNA) was generated from RNA using High-Capacity RNA-to-cDNA Kit® [Thermo Fisher Scientific®, Waltham, MA, USA] and 100 ng cDNA was subsequently used in 20 µl qRT-PCR reactions with TaqMan® Gene Expression Assays [Applied Biosystems®, Foster City, CA, USA]. *ACTB* or *18S* was used as endogenous control in 1:10 dilutions from the cDNA sample used for target gene analysis. Reactions were performed in triplicates on a QuantStudio™ 7 Flex System [Applied Biosystems®] using the standard thermal cycler protocol.

The following TaqMan® Gene Expression Assays were used:

HES1: Hs00172878_m1

CCL4: Hs01092201_m1

CCL3: Hs00234142_m1

CCL2: Hs00234140_m1

Fold changes were calculated towards the mean ΔCt-value of all reference samples.

Liquid chromatography tandem-mass spectrometry (LC-MS/MS) based phosphoproteomics

Briefly, cells were treated with rituximab or obinutuzumab (5 µg/ml) for 0h, 1h or 24 h in biological triplicates. Cells were lysed, protein concentrations normalized, proteins reduced and alkylated prior to tryptic digest. Subsequently, digests were desalted and underwent TiO2

based phospho-enrichment. Reconstituted samples were analyzed twice using an automated data-dependent acquisition on a Q-Exactive Plus mass spectrometer (Thermo Scientific). Peptide identification was conducted using Mascot Distiller 2.3.2 / Mascot Daemon 2.5 and label-free peptide quantification using in-house developed Peak statistics calculator (PESCAL) software. Extracted ion chromatograms were generated for each phosphopeptide ion and quantification values calculated by measuring areas under the curve. Analytical replicated (N=2) were averaged for each biological replicate (N=3). Quantified peptide ions that possessed the same phosphorylation site were combined. Differences in phosphorylation levels between each treatment group and the untreated control group were tested for significance by unpaired non-parametric t-tests conducted in GraphPad Prism version 8.1.1. Kinase activity was inferred by kinase substrate enrichment analysis (KSEA) as previously described (Casado et al., 2013). Pathway enrichment analysis was conducted through the Database for Annotation, Visualization and Integrated Discovery (DAVID) v6.8 (Huang da et al., 2009a; Huang da et al., 2009b). LC-MS/MS raw data is publicly available (PRIDE ID PXD023572).

A more detailed description of sample preparation, LC-MS/MS analysis and data processing is provided below.

Mass spectrometry sample preparation

1 x 10^7 SU-DHL-4 cells were either treated with rituximab or obinutuzumab for 1 or 24 hours, using a monoclonal antibody concentration of 5 μg/ml. Untreated controls were run in parallel. All conditions were run in biological triplicates.

Post antibody treatment, cell lysis, protein normalization, digestion, and phosphopeptide-enrichment were performed as previously described[43]. Briefly, cells were washed three times in cold PBS supplemented with 1 mM Na_3VO_4 and 1 mM NaF. Cells were then lysed in urea buffer (8 M urea in 20 mM HEPES pH 8.0 supplemented with 1 mM

Na_3VO_4, 1 mM NaF, 1 mM $Na_4P_2O_7$ and 1 mM sodium β-glycerophosphate). Cell extracts were sonicated (10 cycles of 30 sec on and 40 sec off; Bioruptor® Plus, Diagenode, Liege, Belgium). Insoluble material was removed by centrifugation at 15,000 rpm for 10 min at 4 ˚C.

Protein was quantified by bicinchoninic acid (BCA) assay (Pierce™ BCA Protein Assay Kit, Thermo Fisher Scientific), and 350 µg of protein was reduced and alkylated by sequential incubation with 10 mM DTT and 16.6 mM iodoacetamyde for 40 minutes. Urea concentration was diluted to 1.44 M with 20 mM HEPES (pH 8.0), prior to the addition of preconditioned trypsin beads as per manufacturers specifications [(Immobilized Trypsin, TPCK Treated, Thermo Fisher Scientific)] and incubation for 18h at 37 ˚C. Trypsin beads were removed by centrifugation at 2,000 x g for 5 min at 4˚C.

Peptide solutions were desalted using 10 mg OASIS-HLB cartridges (Waters, Manchester, UK). Cartridges were activated with ACN (100%) and equilibrated with 1.5 mL washing solution (1% ACN, 0.1% TFA). After loading the samples, cartridges were washed with 1 mL of washing solution. Peptides were eluted with 500 µL of glycolic acid buffer (1 M glycolic acid, 50% ACN, 5% TFA).

To enrich phosphopeptides, sample volumes were normalised to 600 µL using glycolic acid buffer (1 M glycolic acid, 80% ACN, 5% TFA) and 50 µL of TiO_2 beads [(50% slurry in 1% TFA), GL Sciences, Shinjuku, Tokio, Japan] were added to the peptide mixture and incubated for 5 min at room temperature with agitation and centrifuged for 30 s at 1,500 x g. Pelleted TiO_2 beads were then loaded into an empty PE-filtered spin-tip (Glygen, Columbia, MD, USA) prewashed with ACN and packed by centrifugation at 1500 x g for 3 min. The remaining supernatants were then applied to respective spin tips by centrifugation at 1,500 x g for 2 min, and then sequentially washed by 3 min centrifugation at 1,500 x g with glycolic acid buffer, 100 mM ammonium acetate (25% ACN) and 10% ACN. For phosphopeptide recovery,

peptides were eluted with 5% ammonium water. Eluents were dried in a speed vac and peptide pellets stored at -80 ˚C.

LC-MS/MS analysis

For LC-MS/MS analysis, peptides were resuspended in 12 µL of reconstitution buffer (97% H20, 3% ACN, 0.1% TFA, 50 fmol/µl-1 enolase peptide digest), sonicated for 1 min at room temperature and placed in the autosampler (4 °C) until analyzed. Each sample was analyzed twice (4 µl injections). The LC-MS/MS system consisted of a nanoflow ultrahigh pressure liquid chromatography system (UltiMate™ 3000 RSLCnano, Dionex, Sunnyvale, CA, USA) coupled to a Q-Exactive Plus (Thermo Fisher Scientific).

The LC system used mobile phases A (3% ACN: 0.1% FA) and B (100% ACN; 0.1% FA). Peptides were loaded onto a µ-pre-column (Thermo Fisher Scientific) and separated in an analytical column (EASY-Spray, Thermo Fisher Scientific). The gradient was 1% B for 5 min, 1% B to 35% B over 60 min. Following elution, the column was washed with 85% B for 7 min and equilibrated with 3% B for 7 min at a flow rate of 0.25 µL/min. Peptides were nebulized into the online connected Q-Exactive Plus system operating with a 2.1s duty cycle. Acquisition of full scan survey spectra (m/z 375-1,500) with a 70,000 FWHM resolution was followed by data-dependent acquisition in which the 15 most intense ions were selected for HCD (higher energy collisional dissociation) and MS/MS scanning (200-2,000 m/z) with a resolution of 17,500 FWHM. A 30 sec dynamic exclusion period was enabled with an exclusion list with 10 ppm mass window. Overall duty cycle generated chromatographic peaks of approximately 30 sec at the base, which allowed the construction of extracted ion chromatograms (XICs) with at least 10 data points.

Peptide identification and quantification

Mascot Distiller 2.3.2 was used to fit an ideal isotopic distribution to the MS/MS data to maximize peptide identification. Mascot Daemon 2.5 search engine was used to match peaks to peptides in proteins present in the Uniprot/SwissProt Database (human species). The process was automated with Mascot Daemon 2.5.0. Mass tolerance was set to ± 10 ppm, with variable modifications phospho (ST), phospho (Y), gln→pyro-glu (N-term Q) and oxidation (M) included in the search. Carbamidomethyl (C) as fixed modification. Trypsin was selected as digestion enzyme and 2 miss cleavages were allowed. Sites of modification were reported when they had delta scores >10.

Peptide and subsequent protein quantification was achieved using in-house developed PESCAL (Peak statistics calculator) software (Cutillas, 2017). PESCAL constructs extracted ion chromatograms (XICs) for each peptide identified with the MASCOT search engine. With each constructed XIC, peak heights could be calculated. These peptide peak heights were then normalized to the sum of the intensities for each individual sample and the average fold change between conditions could be determined. Statistical significance between conditions was considered significant when the Student T-Tests produced $P < 0.05$. Further data processing and analysis was conducted within Microsoft Excel (2007/2010) or R (v3.3.2/v3.4.1 – reshape2, ggplot2, gplots, readXL, Hmisc and limma packages).

Kinase substrate enrichment analysis (KSEA) was performed as described before (Casado et al., 2013). Briefly, peptides differentially phosphorylated between a set of samples were grouped into substrate sets known to be phosphorylated by a specific kinase as annotated in the PhosphoSite, Phospho.ELM, and PhosphoPOINT databases (Hornbeck et al., 2015; Dinkel et al., 2011; Yang et al., 2008). To infer enrichment of substrate groups across sets of samples the hypergeometric test was used, followed by Benjamini Hochberg multiple testing correction.

Supplemental references

Casado, P., Rodriguez-Prados, J. C., Cosulich, S. C., Guichard, S., Vanhaesebroeck, B., Joel, S. & Cutillas, P. R. (2013). Kinase-substrate enrichment analysis provides insights into the heterogeneity of signaling pathway activation in leukemia cells. Sci Signal 6**,** rs6.

Cutillas, P. R. (2017). Targeted In-Depth Quantification of Signaling Using Label-Free Mass Spectrometry. Methods Enzymol 585**,** 245-268.

Dinkel, H., Chica, C., Via, A., Gould, C. M., Jensen, L. J., Gibson, T. J. & Diella, F. (2011). Phospho.ELM: a database of phosphorylation sites--update 2011. Nucleic Acids Res 39**,** D261-267.

Epstein, A. L. & Kaplan, H. S. (1979). Feeder layer and nutritional requirements for the establishment and cloning of human malignant lymphoma cell lines. Cancer Res 39**,** 1748-1759.

Hornbeck, P. V., Zhang, B., Murray, B., Kornhauser, J. M., Latham, V. & Skrzypek, E. (2015). PhosphoSitePlus, 2014: mutations, PTMs and recalibrations. Nucleic Acids Res 43**,** D512-520.

Huang Da, W., Sherman, B. T. & Lempicki, R. A. (2009a). Bioinformatics enrichment tools: paths toward the comprehensive functional analysis of large gene lists. Nucleic Acids Res 37**,** 1-13.

Huang Da, W., Sherman, B. T. & Lempicki, R. A. (2009b). Systematic and integrative analysis of large gene lists using DAVID bioinformatics resources. Nat Protoc 4**,** 44-57.

Stacchini, A., Aragno, M., Vallario, A., Alfarano, A., Circosta, P., Gottardi, D., Faldella, A., Rege-Cambrin, G., Thunberg, U., Nilsson, K. et al. (1999). MEC1 and MEC2: two new cell lines derived from B-chronic lymphocytic leukaemia in prolymphocytoid transformation. Leuk Res 23**,** 127-136.

Yang, C. Y., Chang, C. H., Yu, Y. L., Lin, T. C., Lee, S. A., Yen, C. C., Yang, J. M., Lai, J. M., Hong, Y. R., Tseng, T. L., et al. (2008). PhosphoPOINT: a comprehensive human kinase interactome and phospho-protein database. Bioinformatics 24**,** i14-20.
